# Transcriptome Analysis Reveals the Role of GA_3_ in Regulating the Asynchronism of Floral Bud Differentiation and Development in Heterodichogamous *Cyclocarya paliurus* (Batal.) Iljinskaja

**DOI:** 10.3390/ijms23126763

**Published:** 2022-06-17

**Authors:** Yinquan Qu, Xiaolin Chen, Xia Mao, Peng Huang, Xiangxiang Fu

**Affiliations:** Co-Innovation Center for Sustainable Forestry in Southern China, Nanjing Forestry University, Nanjing 210037, China; qyquan@njfu.edu.cn (Y.Q.); chenxl0811@163.com (X.C.); mx1991@jsafc.edu.cn (X.M.); hp0610313@163.com (P.H.)

**Keywords:** *Cyclocarya paliurus*, heterodichogamy, floral bud differentiation, GA-related DEGs

## Abstract

*Cyclocarya paliurus* is an important medical plant owing to the diverse bioactive compounds in its leaves. However, the heterodichogamy with female and male functions segregation within protandry (PA) or protogyny (PG) may greatly affect seed quality and its plantations for medicinal use. To speculate on the factor playing the dominant role in regulating heterodichogamy in *C. paliurus*, based on phenotypic observations, our study performed a multi comparison transcriptome analysis on female and male buds (PG and PA types) using RNA-seq. For the female and male bud comparisons, a total of 6753 differentially expressed genes (DEGs) were detected. In addition, functional analysis revealed that these DEGs were significantly enriched in floral development, hormone, and GA-related pathways. As the dominant hormones responsible for floral differentiation and development, gibberellins (GAs) in floral buds from PG and PA types were quantified using HPLC-MS. Among the tested GAs, GA_3_ positively regulated the physiological differentiation (S0) and germination (S2) of floral buds. The dynamic changes of GA_3_ content and floral morphological features were consistent with the expression levels of GA-related genes. Divergences of GA_3_ contents at S0 triggered the asynchronism of physiological differentiation between male and female buds of intramorphs (PA-M vs. PA-F and PG-F vs. PG-M). A significant difference in GA_3_ content enlarged this asynchronism at S2. Thus, we speculate that GA_3_ plays the dominant role in the formation of heterodichogamy in *C. paliurus*. Meanwhile, the expression patterns of GA-related DEGs, including *CPS*, *KO*, *GA20ox, GA2_OX_*, *GID1*, and *DELLA* genes, which play central roles in regulating flower development, coincided with heterodichogamous characteristics. These results support our speculations well, which should be further confirmed.

## 1. Introduction

*Cyclocarya paliurus* (Batal.) Iljinskaja is the sole species in the genus *Cyclocarya* Iljinskaja (Juglandaceae) [1]. It is mainly distributed at an altitude of 420–2500 m in subtropical mountainous areas of Jiangxi, Zhejiang, Anhui, Fujian, Hubei, Sichuan, Guizhou, Guangxi, etc. [2]. *C. paliurus* is generally known as the sweet tea tree, and its leaves are used to cure fever, detoxify, and alleviate pain [3]. Moreover, it was widely used in ancient China to make a medicinal tea [4]. Recently, various studies have found that many potentially bioactive compounds in the leaves of *C. paliurus*, including polysaccharides, proteins, minerals, phenolic compounds, vitamins, saponins, sugars, triterpenoids, and amino acid have beneficial effects on antihypertensive activity, hypoglycemic activity, hypolipidemic activity, and antioxidant activity [5,6]. There is an urgent need to cultivate *C. paliurus* for leaf production to fulfill the need for its medicinal use.

Owing to the lack of advancement in asexual reproduction technology, sexual reproduction is still the primary means of reproduction. Nevertheless, asynchronous flowering exists in heterodichogamous *C. paliurus*, resulting in the low seed setting rate and high seed empty rate in its natural state [7] to limit resource expansion [8]. As a transitional type from dichogamy to dioecy, heterodichogamy possesses two complementary morphs in the monoecious population, including protogyny (PG) and protandry (PA). For PG, the stigma is receptive before pollen dispersal; in PA, pollen scatter occurs before stigma maturation. Additionally, opposite sexual flowers between the PA and PG types at two flowering phases are synchronized. For instance, when the pollen in PA individuals disperses, the stigma in PG ones is in the receptivity state [9,10,11,12].

The synchronism of male and female flowering originates from different processes of floral bud differentiation and development. The complex process of floral development arises in response to the integration of signals from the external environment and internal factors, such as temperature, light, nutrients, and hormones [13,14]. As the signaling molecules, hormones are the influential factors involved in the regulation of vegetative and reproductive growth in the plant kingdom [15]. In addition, a complex regulatory network associated with cytokinin (CK), auxin (IAA), abscisic acid (ABA), and gibberellin (GA) plays an important role in floral bud differentiation in many species [16,17]. Generally, CK, ABA, and IAA are considered as positive regulators that promote flower bud growth [18,19,20]. For example, using exogenous GA promoted stem elongation and stimulated early flowering in *Agapanthus praecox* ssp. *orientalis* [21]. Cecich demonstrated that the timing of gibberellin A_4/7_ spray application affected flowering response in white spruce (*Piceaglauca* (Moench) Voss) [22]. Moreover, spraying GA inhibitor on *Carica papaya* L. could induce carpel development in the male plant [23]. Exogenous GA treatment on cucumber (*Cucumis sativus*) and spinach (*Spinacia oleracea*) induced male expression [24]. In contrast, several studies have shown that low GA content is beneficial for flower bud differentiation in apples (*Malus pumila*) [25], ginkgo (*Ginkgo biloba* L.) [26], and rose (Rosa × wichurana) [27].

Various studies have stated the effects of GAs on homogamy plants, including dioecism (such as *ginkgo*) and monoecism (such as apple). However, the regulating mechanism of GAs remains unknown in asynchronous floral differentiation and developmental processes in dichogamous plants. Heterodichogamy, a special type of dichogamy, was found in Magnoliaceae [28], Calycanthaceae [29], Ranunculaceae [30], Oleaceae [31], Celastraceae [32], and half of the species in Juglandaceae (*Juglans regia* L. [33], *Juglans mandshurica* Maxim [34], *C. paliurus* [8], etc.). As described on phenological observation, two separated flowering phases in these plants occurred during the flowering period: the first phase was the maturation of the male flower in PA and female in PG, while the maturation of female in PA and male in PG was found in the second phase [35]. Such a phenomenon was also noticed in medicinal *C. paliurus* [12].

Due to the solid basis and application necessary in *C. paliurus*, we took this species as an example, aiming to investigate the following puzzles: (1) do GAs (and if so, which GAs) play a role in regulating two sexual floral differentiations and development; and (2) if so, how do GAs regulate the asynchronism of opposite sexual flowers of intramorphs (PA or PG). In this study, we not only describe the effects of GAs on flower bud differentiation in heterodichogamous plants and its regulation mechanism, but also provide an effective method of improving seed (fruit) yield for high-efficiency cultivation.

## 2. Results

### 2.1. Phenotypic Characterization

**Figure 1:**
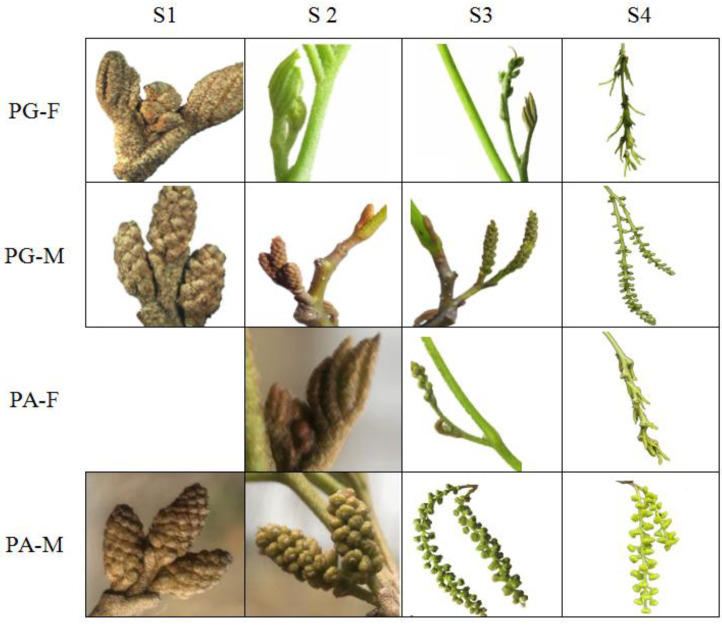
Comparison of phenotypic characteristics in four floral buds during different stages. PG-F: female floral buds of PG type; PG-M: male floral buds of PG type; PA-F: female floral buds of PA type; PA-M: male floral buds of PA type.

Different stages of floral buds in *C. paliurus* were selected for RNA-Seq analysis based on morphological changing. At S0, all flower buds were not visible. At S1, although PA-F buds enclosed by leaf buds were not visible, male and PG-F buds were clearly differentiated. At S2, all the flower buds began to protrude, except PA-F. At S3, feathery stigma formed in PG-F, while this was not observed in PA-F; PA-M turned from turquoise to yellow-green with anther dehiscence, while PG-M still remained turquoise. At S4, the feathery stigma of PG-F opened and the mature pollen of PA-M was released from the dehisced anther; the stigma of PA-F was not fully formed, and the anther of PG-M was slowly dehiscent (Figure 1).

### 2.2. Sequencing, Assembly, and Annotation of C. paliurus

RNA-seq was performed on the Illumina Novaseq platform. Four flower buds (PG-F, PG-M, PA-F, and PA-M) at five stages (S0, S1, S2, S3, and S4) and leaves were used for transcriptome profiling. In total, more than 64 million raw reads were obtained. After quality evaluation and filtration, the number of clean reads ranged from 64,141,024 to 79,962,072 with an average of 73,855,021 and the average value of Q30 was 94.81%, indicating that the clean reads were of high quality. After alignment with the rubber tree genome sequence, more than 94% of the clean reads were mapped to the genome and paired-end reads were aligned to 35,221 *C. paliurus* annotated gene models (Table S1).

### 2.3. Identification of Differentially Expressed Genes (DEGs) during Floral Bud Differentiation and Development

**Figure 2:**
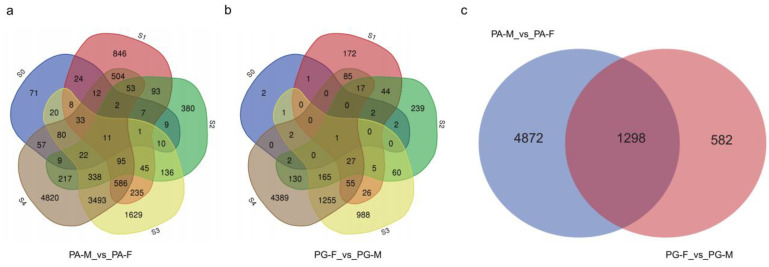
Venn diagrams of DEGs in comparisons: (**a**) DEGs in PA-M vs. PA-F comparison; (**b**) DEGs in PG-F vs. PG-M; and (**c**) DEGs in two comparisons.

Two comparisons of three floral buds (PA-M vs. PA-F, PG-F vs. PG-M) detected a total of 6753 DEGs that were consistently upregulated or downregulated in at least two stages (Figure 2). Comparatively, the number of DEGs in PA-M vs. PA-F (6170) were more than in PG-F vs. PG-M (1880) (Figure 2).

### 2.4. GO Functional Annotation and KEGG Pathway Enrichment of DEGs

**Figure 3:**
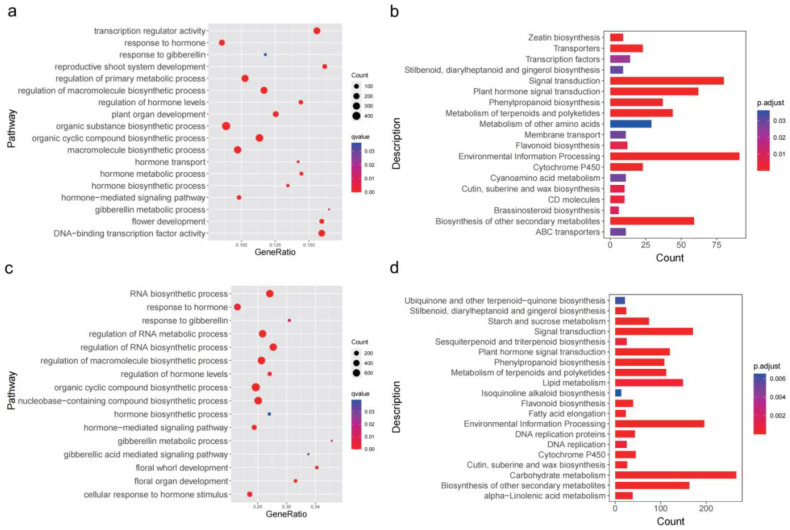
GO and KEGG enrichment analysis of DEGs in PG-F vs. PG-M (**a**,**b**) and PA-M vs. PA-F (**c**,**d**) during five stages of floral buds in *C. paliurus*.

For the PG-F vs. PG-M comparison, GO functional annotation showed that most of the DEGs during the five stages were enriched in a series of biological processes involved in flower and organ development, hormone biosynthetic and metabolic processes, and GA signaling pathways (Figure 3a). Moreover, KEGG pathway enrichment revealed plant hormone signal transduction, environmental information processing, signal transduction, cytochrome P450, and the metabolism of terpenoids (Figure 3b). Notably, many of the up-regulated DEGs (371/1880 in PG-F) were related to hormone (GA) biosynthesis and signaling pathways.

For the PA-M vs. PA-F comparison, the functional analysis revealed that these DEGs were also enriched in floral development, hormone, and GA-related pathways (Figure 3c,d). Similarly, a large number of up-regulated DEGs (893/6170 in PA-M) were associated with hormone biosynthesis and metabolic processes and signal transduction. These results indicated that hormones, especially GA, may play an important role in contributing to the heterodichogamy morphs with asynchronous flowering.

### 2.5. DEGs Related to Gibberellin in Biosynthesis and Regulatory Pathways

DEGs in two comparisons were both significantly enriched in hormone and gibberellin biosynthesis, metabolic, and signaling pathways. Moreover, a series of ABCE module genes, such as AP3, PI, AG, and SEP, could be effectively activated by GA and play key role in regulating floral stamen and pistil morphogenesis [36,37]. Hence, we especially focused upon the DEGs associated with gibberellin (GA) biosynthesis and signal pathways. 

**Figure 4:**
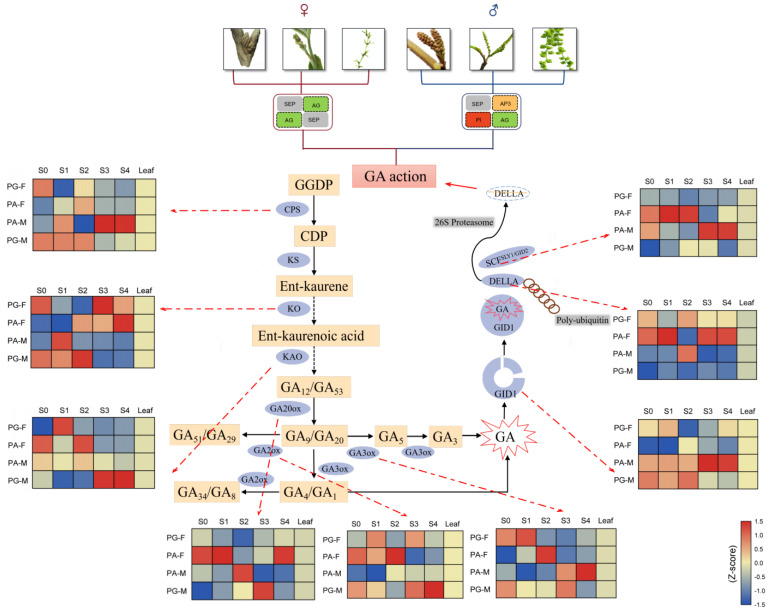
Heatmaps of DEGs expression related to gibberellin in biosynthesis and regulatory pathway. Average expression was applied for multi-copy gene.

For the PG-F vs. PG-M comparison, many genes that are involved in GA biosynthesis, such as CPS (ent-copalyl diphosphate synthase), KO (ent-kaurene oxidase), GA20ox (GA 20-oxidase), GA2ox (GA 2-oxidase), and GID1 (gibberellin receptor), were highly expressed in PG-M at the S2 stage. However, DELLA genes, which negatively modulate GA signaling via binding to GAs and play important functions in inhibiting vegetative and reproductive growth, were lowly expressed in PG-F at S2 (Figure 4).

For the PA-M vs. PA-F comparison, GA2ox and DELLA genes were highly expressed in PA-M at S2, but lowly expressed at S0. However, a series of genes including CPS, KO, KAO (ent-kaurenoic acid oxidase), and GA3ox (GA 3-oxidase) were highly expressed in PA-F at S2 (Figure 4). Taken together, the heterodichogamy within PA or PG might be associated with the floral induction pathway promoted by GA signaling.

### 2.6. GA_3_ Plays a Critical Role in Floral Differentiation and Development

The effect of endogenous GAs on floral differentiation and development was investigated; the evolving patterns of four GAs (bioactive GA_1_, GA_3_, GA_4_ and GA_7_) contents are depicted in Table 1. The analysis of variance showed that there was a significant difference in the contents of GAs during the process of male and female flower differentiation and development in two mating types (*p* = 0.049). Among the four GA types, only small contents of GA_1_, GA_4_, and GA_7_ were detected, and their fluctuations changed little across the floral developmental process. Comparatively, the contents of GA_4_ and GA_7_ in floral buds were negligible, although the content of GA_1_ was higher than that of GA_4_ and GA_7_. However, the maximum of GA_3_ in four floral buds was much higher than that of the other three GA types, varying from 40.37 ng/g FW to 116.50 ng/g FW at the S0 and S2 stages, with the minimum decreasing abruptly to trace amounts at the S3 and S4 stages (Table 1). So, the ranges of GA_3_, varying from 35.81 ng/g FW to 113.97 ng/g FW, fluctuated greatly across the five stages, indicating its prominent effects on regulating floral differentiation and development. In summary, the flower bud differentiation and development in *C. paliurus* greatly depend on the content changes of GA_3_ rather than GA_1_, GA_4_, and GA_7_. Therefore, the dynamics of GA_3_ content were separately analyzed in the following study.

### 2.7. Dynamic Patterns of GA_3_ Content in Male and Female Floral Buds

#### 2.7.1. PG Mating Type

**Figure 5:**
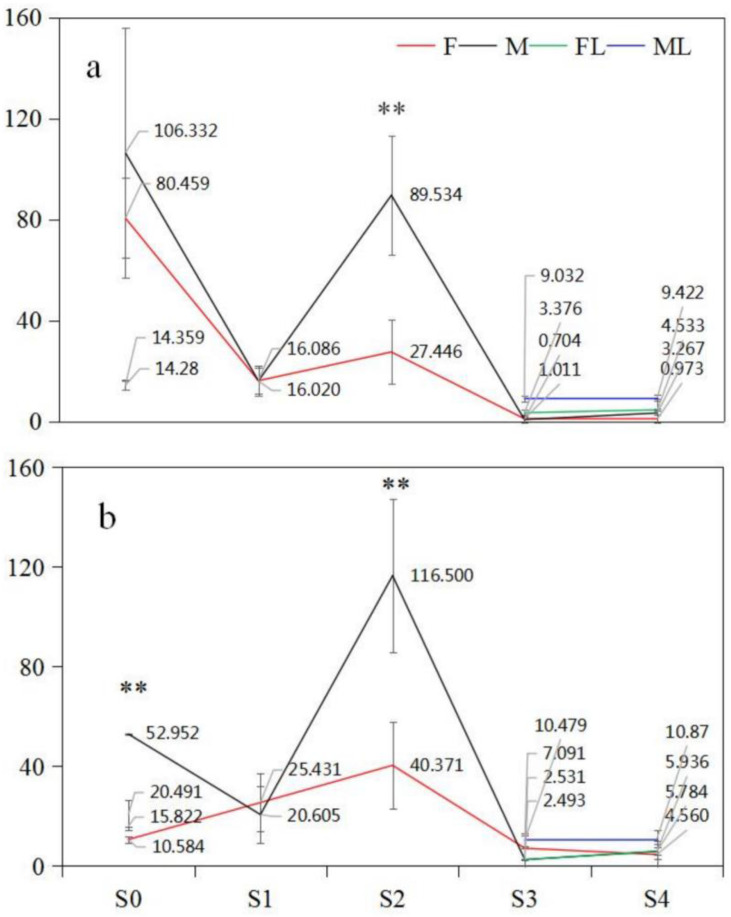
Changing patterns of GA_3_ content in male and female buds and surrounding leaves in two mating types of C. paliurus: (**a**) protogyny (PG) and (**b**) protandry (PA); (F) female flower buds, (M) male flower buds, (F, L) leaves surrounding female flower buds, (M, L) leaves surrounding male flower buds; ** shows the significant difference of GA_3_ contents between F and M at the same stage (*p* < 0.05).

For female floral buds (PG-F), GA_3_ contents were higher at both S0 and S2 than other stages; particularly at S0, the content reached the largest value (80.46 ng/g FW), while the lowest level (<16.09 ng/g FW) was measured at other stages (S1, S3, and S4). For male floral buds (PG-M), a similar changing pattern of GA_3_ content to that in PG-F was observed, with higher values at S0 and S2 but lower values at S1, S3, and S4. Remarkably, the contents in PG-M were higher at S0 (106.33 ng/g FW) and S2 (89.53 ng/g FW) than in PG-F at those stages (Figure 5a). These results indicated that GA_3_ could positively regulate the physiological differentiation (S0) and germination of male and female floral buds (S2) in PG, and make no difference at other phases; this was also supported by the significant differences of GA_3_ contents in male/female flower buds over various stages (*p* < 0.05). Multiple comparisons revealed no significant differences between males and females at S0, suggesting that GA_3_ could regulate both sexual floral physiological differentiations. However, the difference of GA_3_ content between two floral buds at S2 was significant (*p* < 0.05), showing that a higher GA_3_ content is needed for the germination of male floral buds than for female ones. All findings were confirmed by their corresponding morphological differences: differentiated male floral buds were visible but differentiated female buds were invisible at S0. Moreover, the onset of germination of male buds was earlier and at a faster rate than that of females at S2 (Figure 5).

#### 2.7.2. PA Mating Type

In PA, GA_3_ content in male and female buds at all stages displayed identical patterns to those in PG, except for a lower value in PA-F at S0. These dynamics correlated well with the characteristics of sexual floral bud differentiation and growth in PA, where the process of male floral buds was prior to that of female buds (Figure 5b). The low GA_3_ content of female floral buds at S0 was entirely consistent with that, as their physiological distinction in S0 was not completed until S2 in the following year (Figure 1). At the S0 and S2 stages, GA_3_ variation in two sexual floral buds confirmed that the quantity of GA_3_ could determine the differentiation in floral buds and germination. Also, the higher GA_3_ content in male than in female buds further suggests that the germination of male floral buds could be more dependent on GA_3_ content than female ones.

Likewise, the low GA_3_ content of all leaves around male and female flower buds in both mating types indicates that the leaves may not be the source of GA_3_ for flower bud differentiation and growth (Figure 5).

### 2.8. Real Time Quantitative PCR Validation of RNA-seq Results

**Figure 6:**
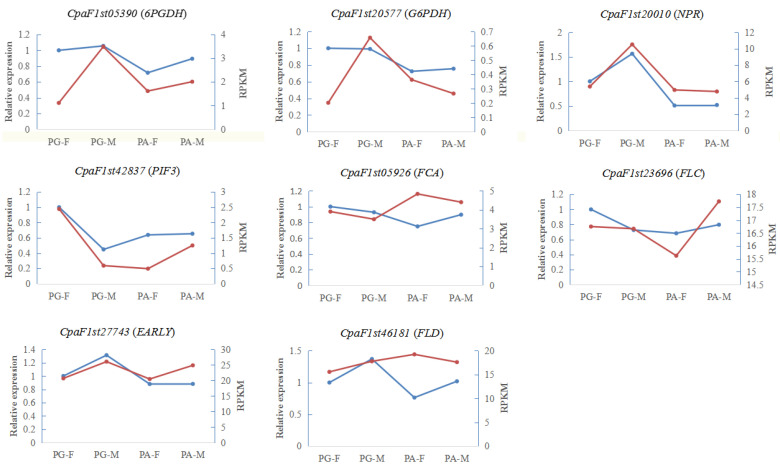
Comparisons of expression levels of eight genes obtained by qRT-PCR and RNA-seq analysis in different floral buds during germination in *C. paliurus*. All qRT-qPCRs for genes were conducted in triplicate, with two repeats per experiment. Red line represents the relative expression and blue line represents the RPKM.

Eight candidate genes were selected for validation by qRT-PCR in floral buds at S2 (Figure 6), including *CpaF1st05390* (*6PGDH*), *CpaF1st20577* (*G6PDH*), *CpaF1st20010* (*NPR*), *CpaF1st42837* (*PIF3*), *CpaF1st05926* (*FCA*), *CpaF1st23696* (*FLC*), *CpaF1st27743* (*EARLY*), and *CpaF1st46181* (*FLD*). Generally, the relative expression of selected genes was consistent with the RPKM values from RNA-seq, indicating the expression profiles of our transcriptomics results were reliable (Figure 6).

## 3. Discussion

Heterodichogamy is described as the presence of two structures that exhibit male and female characteristics at different times (within the same flower). Particularly, the PA type is male prior to female and the PG type is female prior to male, and two flowering stages appear during the flowering season. Hence, flowering asynchronization emerges not only between the male and female flower of intramorphs (such as PA-F and PA-M), but also the same sexual flower of intermorphs (such as PG-F and PA-F). This phenomenon has been noticed in many heterodichogamous plants, particularly in Juglandaceae species, such as *Juglans regia* L. [35], *J. mandshurica* [38], *Carya illinoinensis* [39], *Platycarya strobilacea* [11], and *C. paliurus* [12]. However, our study tries to shed light on the puzzle of what regulates floral buds to realize heterodichogamy in these plants. 

For male and female flowers of intramorphs, GA-related genes may regulate the asynchronization by perceiving the position differences of two sexual buds. As stated by Mao et al. [12], in *C. paliurus* female buds grow at the apex of the growing shoot and male buds locate at the lateral short branch; the expression of GA-related DEGs in male and female buds may be triggered under the genetic control depending on the mating type. Based on the analysis of differentially expressed genes, 371 enriched DEGs in PG-F vs. PG-M and 893 DEGs in PA-M vs. PA-F related to gibberellin biosynthesis and signal transduction pathways further confirmed the GA_3_ role in regulating floral differentiation in *C. paliurus*. For the differentiation and germination of floral buds at S0 and S2, a series of GA-related genes, including *CPS*, *KO*, *KAO*, *GA20ox*, *GA2ox*, *GA3ox*, *GID1*, *DELLA*, and *SCF*, were differentially expressed, demonstrating the importance of GA_3_ in floral bud differentiation and germination. For instance, as the positive key enzyme genes, *CPS*, *KO*, *KAO*, and *SCF* were significantly down-regulated in the comparison of PA-M vs. PA-F (Figure 4), which is consistent with the phenomenon that the physiological differentiation of female buds was not complete until S2 in PA. However, *DELLA* was significantly up-regulated in the comparison of PA-M vs. PA-F at S2, confirming the functions of DELLA proteins on inhibiting vegetative and reproductive growth [40,41]. For the PG-F vs. PG-M comparison, *GID1* showed a higher expression level and *DELLA* showed a lower level in PG-M at S2, which indirectly supported that GA was more necessary for male buds germination than female. 

Previous studies have proved that gibberellins have been implicated in coordinating flower differentiation and development by regulating various cellular changes in response to environmental or intrinsic signals. For instance, among GAs, GA_1_, GA_4_, and GA_7_ were reported to partially inhibit floral initiation in *Prunus avium* [42], while bioactive GA_4_ was necessary for female flower development in *Cucumis* [43]; moreover, GA_3_ not only slightly delayed senescence but also affected flower opening in *Iris* × *hollandica* cv. Blue Magic [44]. Furthermore, exogenous GA_3_ application partially restored the inhibitory effects of heavy metals on flowering yield in *Lagenaria siceraria* (Mol.) Standl and *Luffa cylindrica L.* (Roem) [45], and significantly delayed floral induction in ‘Fuji’ apples (*Malus domestica* Borkh.) [46]. In this study, in contrast to other GAs in four types of floral buds (PG-F, PG-M, PA-F, PA-M), GA_3_ content showed significantly higher values with apparent variations over five stages (Table 1), suggesting its critical role in controlling flowering bud differentiation and development. At the S0 stage, a low level of GA_3_ in female buds and a high level in male buds in PA (Figure 5) is in accordance with the anatomical observations (data unpublished), which revealed the differentiated male buds and undifferentiated female buds. On the contrary, the high GA_3_ content of both floral buds in PG (Figure 5) was also consistent with the anatomical findings, which showed differentiated floral buds but female before male buds. 

In addition, higher GA_3_ contents in male over female buds in both the PA and PG types at two stages infers that the activity of male buds could be more dependent on high GA_3_. Collectively, these results from *C. paliurus* are consistent with previous studies (the rhythm in *Kalanchoe* spp., *Brassica rapa* ssp. *chinensis* Makino), that GA_3_ promoted floral induction and germination and even affected the number of days taken to flower [47,48,49]. Quite the reverse, the critical role of GA_3_ in controlling the physiological differentiation in male buds and germination in *C. paliurus* was also verified in *Arabidopsis thaliana*, whose stamen development required higher GA contents than other floral organs (pistil, petal, and calyx) [50]. Moreover, a higher level of GA_3_ in male floral buds at stage 8 (mature pollen) in *Diospyros kaki* Thunb. was considered to promote the development of stamen primordia [51]. Combining GA_3_ content with GA-related DEGs expressions, we deduced that gibberellin response genes potentially play crucial roles in adjusting the asynchronization of the male and female flowers of intramorphs in *C. paliurus*.

## 4. Materials and Methods

### 4.1. Plant Material and Growth Conditions

An experimental plantation of *C. paliurus* was established in Hongya Mountain Forest Farm, Chuzhou, Anhui Province, China (N 31°21′, E 118°58′) in 2013, containing 800 seedlings from a germplasm bank, which were planted in the Baima Experimental Base of Nanjing Forestry University. Voucher specimens were identified by Xin Chen (a taxonomist of Nanjing Forestry University) and deposited in the Silviculture Lab of Nanjing Forestry University (voucher code: 2011GX, 2011SC, 2011HB, 2011ZJ, 2011HN). Nine protogynous (PG) and protandrous (PA) individuals, respectively, were randomly selected and marked for phenological observation and sampling. 

Leaves and floral buds in various protandry (PA) and protogyny (PG) individuals were collected at five different stages of floral differentiation and development including (1) S0, the physiological differentiation period (26 May 2019); (2) S1, the dormancy period (23 December 2019); (3) S2, the germination period (15 March 2020); (4) S3, the inflorescence elongation period (18 April 2020); and (5) S4, the maturation period (28 April 2020). Among them, only male and female floral buds from two mating types were collected as experiment materials at S1, S2, and S3, because of their deciduous characteristic. However, during the other two phases, floral buds and leaves were harvested for GAs content determination. Leaves at S4 and floral buds at five stages were collected for transcriptome sequencing. All flower buds were invisible during physiological differentiation (S0), so the flower buds were harvested based on last year’s position on the branch and the duration of morphological observation was from the dormancy stage (S1) to the maturation period (S4). Samples of complementary gender in each mating type were harvested from three individuals and combined as one independent biological replicate, and the total of nine trees were grouped as three replicates. Collectively, more than 15 male flower buds and at least 45 female flower buds were collected for each replicate, respectively. All samples were preserved in liquid nitrogen and stored at −80 °C for further analysis.

### 4.2. RNA Extraction and Illumina Sequencing

Total RNA was extracted from various tissues using an E.Z.N.A Plant RNA Isolation Kit (OMEGA, Norcross, GA, USA). RNA concentration and purity were determined by a NanoDrop spectrophotometer 2000C (IMPLEN, Westlake Village, CA, USA), and RNA integrity was confirmed by 1.0% agarose gel electrophoresis. The RNAs with the values of OD_260_/OD_280_ within 1.8–2.0 were reverse transcribed to cDNA using a PrimeScript™ RT reagent kit with gDNA Eraser (TaKaRa, Beijing, China), according to the instructions of the manufacturer. Then, the cDNAs were diluted at 1:10 with RNase-free water, and stored at −20 °C for qRT-PCR analyses. All cDNA libraries were loaded onto an Illumina HiSeqTM2000 system (2 × 100 bp read length). Finally, 6 Gb of RNA-seq raw data for each sample was generated, and the quality of raw reads was checked by FastQC [52]. Clean reads were obtained from removing poor-quality sequences (the bases of Q-score < 10%), short sequences (<50 bp), primer sequences, unidentified nucleotides (more than 10%), and adapter sequences.

### 4.3. Assembly and Differentially Expressed Genes (DEGs) Analysis

Paired-end short reads were aligned to the PG-dip *C. paliurus* genome using Hisat2 [53]. StringTie (v.1.3.3b) [54] was used to assemble transcripts and estimate the gene expression levels of each sample. The expected number of FPKM (fragments per kilobase per million reads) fragments mapped were calculated using the RSEM program [55], which was implanted in the Trinity package [56]. Moreover, the DESeq2 R package [57] was applied to identify the DEGs. In multiple analyses, the threshold of the *p*-value was calculated by the false discovery rate (FDR). If the FDR was less than 0.05, and the absolute value of log_2_ (fold change) was more than 1, the gene expression differences were considered to be significant.

The Gene Ontology (GO) functional annotation and Kyoto Encyclopedia of Genes and Genomes (KEGG) pathway analysis were performed using the clusterProfiler R package [58].

### 4.4. Measurement of GAs Content

A UPLC-MS/MS system (UPLC, SHIMADZU Nexera X2; MS/MS, Applied Biosystems 4500 Q TRAP) was used to determine the content of four types of GAs (bioactive GA_1_, GA_3_, GA_4,_ and GA_7_) at different developmental stages. 

Standards (gibberellin, GA_3_) were purchased from Sigma-Aldrich (St. Louis, MO, USA), while MeOH and ACN were purchased from Merck (Darmstadt, Germany). *C. paliurus* vacuum freeze-dried floral buds and leaves were ground into powder (30 Hz, 5 min), and 500 mg of powder was extracted with 1.5 mL of methanol/water/methanoic acid solution (15:4:1, V/V/V). The extract was vortexed every 30 min and stored at 4 °C for one night. After centrifugation (rotating speed 12,000 rpm, 10 min), the supernatants were filtered with a microporous membrane (0.22 µm pore size) and collected for UPLC-MS/MS analysis.

The UPLC operation parameters were as follows: chromatographic separation was carried out using a Waters ACQUITY UPLC HSS T3 (100 mm × 2.1 mm, 1.7 μm) and the mobile phase consisted of ultrapure water (A) and acetonitrile (B), which both contained 0.1% formic acid. Gradient elution was as follows: the original B ratio was 5%; the B ratio linearly increased to 70% within 3 min and was maintained for 3 min; and the B ratio decreased to 5% from 6.1 to 8 min and was maintained for 1.9 min. The flow rate was kept at 0.2 mL/min, temperature at 40 °C, and injection volume was 2 µL.

### 4.5. Quantitative Real Time PCR (qRT-PCR) Analysis

Eight unigenes related to floral bud differentiation and development, as well as the hormone-mediated signaling pathway, were selected for qRT-PCR analysis. Moreover, 18S ribosomal RNA as an internal control and the cDNA Synthesis Kit for reverse transcription were selected according to Chen [59]. Proprietary software with Quant Studio 6 (Life Technologies, Carlsbad, CA, USA) and GoTaq^®^ qPCR Master Mix (PROMEGA, Cat.No.A6002) were used for RT-qPCR reaction. The reaction system was as follows: 2× MasterMix 10 µL, Primer F 1 µL(10 μM), Primer R 1 µL(10 μM), cDNA 1 µL, and nuclease-free water up to 20 µL. The reaction procedure and methods of relative gene expression calculation were presented by Chen [59].

## 5. Conclusions

This study reveals that hormones, especially GA_3_, may play an important role in contributing to the heterodichogamy morphs with asynchronous flowering in *C. paliurus*. The DEGs during five stages were significantly enriched in a series of biological processes involved in flower and organ development, hormone biosynthetic and metabolic processes, and GA signaling pathways. GA_3_ content showed a significant role in controlling flowering bud differentiation and development. Our results can provide the basis for further understanding the molecular mechanisms for gibberellin regulating floral bud differentiation, development, and their asynchronism in heterodichogamous plants.

## Figures and Tables

**Table 1:** Effects of GA content on floral bud differentiation and development in *C. paliurus*. * Range means the difference between the maximum and the minimum in the same type of floral bud.

GA Type	Floral Bud	Maximum		Minimum		Range * (ng/g FW)
		Content (ng/g FW)	Occurred Stage	Content (ng/g FW)	Occurred Stage	
GA_1_	PA-F	1.65	S1	0.29	S2	1.36
	PA-M	7.19	S0	0.20	S3	6.99
	PG-F	3.03	S0	0.00	S3/S4	3.03
	PG-M	4.05	S0	0.00	S3/S4	4.05
GA_3_	PA-F	40.37	S2	4.56	S4	35.81
	PA-M	116.50	S2	2.53	S3	113.97
	PG-F	80.46	S0	0.97	S4	79.49
	PG-M	106.33	S2	0.70	S3	105.63
GA_4_	PA-F	0.61	S4	0.00	S2	0.61
	PA-M	1.07	S2	0.00	S3/S0	1.07
	PG-F	0.78	S2	0.00	S1/S0	0.78
	PG-M	0.97	S0	0.00	S1/S2	0.97
GA_7_	PA-F	0.00	S0/S1/S2/S3/S4	0.00	S0/S1/S2/S3/S4	0.00
	PA-M	0.33	S4	0.00	S0/S2/S3	0.33
	PG-F	3.27	S0	0.00	S2/S4	3.27
	PG-M	0.70	S2	0.00	S1/S2/S3	0.00

## Data Availability

Sixty transcriptome raw data produced by Illumina NovaSeq 6000 have been deposited in the Genome Sequence Archive (SRA) database (https://ngdc.cncb.ac.cn/gsa/s/yLQq1dFX (accessed on 13 May 2022)) under the accession number CRA004671.

## Supplementary Materials

The following supporting information can be downloaded at: https://www.mdpi.com/article/10.3390/ijms23126763/s1.

## Abbreviations

PA	Protandry
PG	Protogyny
PA-F	Female floral buds from a protandrous plant
PA-M	Male floral buds from a protandrous plant
PG-F	Female floral buds from a protogynous plant
PG-M	Male floral buds from a protogynous plant
GAs	Gibberellins
DEGs	Differentially expressed genes
qRT-PCR	Quantitative real-time polymerase chain reaction
FDR	False discovery rate
FPKM	Fragments per kilobase per million reads
