# Supplementary material for: Transcriptome Analysis Reveals the Role of GA3 in Regulating the Asynchronism of Floral Bud Differentiation and Development in Heterodichogamous Cyclocarya paliurus (Batal.) Iljinskaja
Source: Int J Mol Sci. 2022 Jun 17;23(12):6763. doi: 10.3390/ijms23126763 (PMC9224186; doi:10.3390/ijms23126763)
Supplement: Supplementary file 1 [file ijms-23-06763-s001.zip › ijms-1748015-supplementary.pdf]

Table S1. Summary of Illumina high-throughput sequencing of flower buds and leaves in *C. paliurus*.

Samples	Raw Reads	Clean Reads	GC Content	%>Q30	Mapping Rate
0PG-F1	68,004,502	68,003,118	45.41%	95.04%	94.82%
0PG-F2	74,466,454	74,465,020	45.56%	95.13%	95.61%
0PG-F3	74,418,836	74,417,746	45.26%	95.36%	95.09%
0PG-M1	74,412,908	74,411,918	46.39%	95.04%	95.78%
0PG-M2	75,756,788	75,755,804	49.78%	94.55%	95.67%
0PG-M3	79,560,416	79,559,290	46.76%	95.03%	95.61%
0PA-F1	76,086,216	76,085,202	43.89%	95.35%	95.19%
0PA-F2	78,180,812	78,179,634	43.95%	95.19%	95.82%
0PA-F3	73,627,274	73,626,302	44.27%	95.05%	95.82%
0PA-M1	79,029,184	79,028,030	45.43%	95.08%	95.60%
0PA-M2	75,019,608	75,018,700	45.91%	94.92%	95.37%
0PA-M3	74,319,804	74,318,682	45.53%	95.30%	95.42%
1PG-F1	69,627,986	69,626,648	45.79%	93.31%	95.55%
1PG-F2	77,198,180	77,196,840	45.23%	93.34%	95.63%
1PG-F3	67,767,260	67,766,076	46.49%	93.24%	96.28%
1PG-M1	73,671,124	73,669,832	45.46%	93.29%	95.88%
1PG-M2	70,429,582	70,428,308	45.30%	93.38%	95.08%
1PG-M3	70,834,564	70,833,122	45.57%	93.54%	95.73%
1PA-F1	74,084,266	74,082,882	45.31%	93.28%	95.55%
1PA-F2	78,048,436	78,046,840	45.37%	93.71%	95.06%
1PA-F3	68,537,610	68,536,264	45.29%	93.55%	95.97%
1PA-M1	69,737,848	69,736,498	45.45%	93.50%	95.74%
1PA-M2	72,010,400	72,008,878	45.14%	93.62%	95.48%
1PA-M3	72,244,264	72,242,686	45.65%	94.05%	95.31%
2PG-F1	79,963,950	79,962,072	45.70%	96.74%	95.04%
2PG-F2	74,004,534	74,002,916	45.87%	96.74%	95.06%
2PG-F3	75,322,560	75,321,458	45.78%	95.76%	95.53%
2PG-M1	78,115,814	78,114,650	45.40%	95.81%	96.39%
2PG-M2	78,745,150	78,744,004	45.67%	95.73%	95.65%
2PG-M3	75,466,560	75,465,404	45.58%	95.87%	95.93%
2PA-F1	76,937,230	76,935,950	46.05%	96.01%	95.24%
2PA-F2	79,926,632	79,925,388	45.88%	95.89%	95.27%
2PA-F3	75,428,666	75,427,496	45.85%	95.84%	95.81%
2PA-M1	70,174,622	70,173,540	45.70%	95.69%	95.79%
2PA-M2	70,464,346	70,463,352	45.29%	95.77%	94.84%
2PA-M3	75,958,316	75,957,258	45.41%	95.89%	95.71%
3PG-F1	78,526,474	78,525,262	45.61%	95.83%	95.25%
3PG-F2	73,676,958	73,675,766	45.58%	96.01%	95.49%
3PG-F3	75,092,658	75,091,534	45.70%	95.84%	96.16%
3PG-M1	72,512,266	72,511,202	45.19%	95.86%	95.90%
3PG-M2	70,487,900	70,486,802	45.37%	95.74%	95.81%

3PG-M3	74,871,784	74,870,644	45.81%	95.94%	96.37%
3PA-F1	72,119,304	72,118,166	47.06%	95.74%	96.06%
3PA-F2	76,666,972	76,665,818	45.82%	95.76%	95.74%
3PA-F3	73,149,986	73,148,950	45.53%	95.76%	95.45%
3PA-M1	73,760,456	73,759,372	45.50%	95.87%	95.53%
3PA-M2	65,943,122	65,942,178	45.39%	95.88%	96.54%
3PA-M3	73,845,408	73,844,290	45.41%	95.77%	95.04%
4PG-F1	77,714,188	76,911,266	46.43%	93.29%	95.46%
4PG-F2	77,242,136	76,271,624	45.64%	92.90%	95.29%
4PG-F3	74,371,284	73,516,636	45.90%	93.15%	95.37%
4PG-M1	73,655,482	72,872,008	46.19%	93.48%	96.08%
4PG-M2	73,596,470	72,827,984	45.50%	93.58%	95.76%
4PG-M3	74,567,200	73,789,910	45.24%	93.50%	96.81%
4PA-F1	73,557,428	72,796,720	45.31%	93.70%	96.39%
4PA-F2	74,121,884	73,244,128	45.51%	93.20%	95.49%
4PA-F3	79,355,072	78,545,142	45.22%	93.45%	95.18%
4PA-M1	68,045,536	67,357,670	45.39%	93.74%	95.74%
4PA-M2	72,581,478	71,847,892	45.51%	93.58%	95.40%
4PA-M3	64,840,066	64,141,024	45.27%	93.29%	96.09%
4PG-L1	72,660,793	72,650,258	45.49%	95.83%	95.53%
4PG-L2	75,452,953	74,952,564	45.57%	95.57%	95.58%
4PG-L3	72,578,652	72,426,655	46.10%	95.66%	96.62%
4PA-L1	72,648,236	72,616,469	45.78%	94.35%	95.47%
4PA-L2	75,648,236	74,252,055	45.34%	95.19%	95.60%
4PA-L3	75,236,468	75,233,564	45.55%	95.57%	95.92%
